# Mitochondrial fission regulates germ cell differentiation by suppressing ROS-mediated activation of Epidermal Growth Factor Signaling in the *Drosophila* larval testis

**DOI:** 10.1038/s41598-019-55728-0

**Published:** 2019-12-23

**Authors:** Rafael Sênos Demarco, D. Leanne Jones

**Affiliations:** 1 Department of Molecular, Cell and Developmental Biology, University of California, Los Angeles, Los Angeles, CA 90095 USA; 2 Molecular Biology Institute, University of California, Los Angeles, Los Angeles, CA 90095 USA; 3 Eli and Edythe Broad Center of Regenerative Medicine and Stem Cell Research, University of California, Los Angeles, Los Angeles, CA 90095 USA

**Keywords:** Spermatogenesis, Organelles

## Abstract

Mitochondria are essential organelles that have recently emerged as hubs for several metabolic and signaling pathways in the cell. Mitochondrial morphology is regulated by constant fusion and fission events to maintain a functional mitochondrial network and to remodel the mitochondrial network in response to external stimuli. Although the role of mitochondria in later stages of spermatogenesis has been investigated in depth, the role of mitochondrial dynamics in regulating early germ cell behavior is relatively less-well understood. We previously demonstrated that mitochondrial fusion is required for germline stem cell (GSC) maintenance in the *Drosophila* testis. Here, we show that mitochondrial fission is also important for regulating the maintenance of early germ cells in larval testes. Inhibition of *Drp1* in early germ cells resulted in the loss of GSCs and spermatogonia due to the accumulation of reactive oxygen species (ROS) and activation of the EGFR pathway in adjacent somatic cyst cells. EGFR activation contributed to premature germ cell differentiation. Our data provide insights into how mitochondrial dynamics can impact germ cell maintenance and differentiation via distinct mechanisms throughout development.

## Introduction

Mitochondria are dynamic organelles that undergo constant fusion and fission through the action of dynamin-related GTPases in order to maintain cellular homeostasis^1^. Mitofusins (Mfns) and optic atrophy 1 (Opa1) bring the outer and inner mitochondrial membranes together, respectively, to promote mitochondrial fusion^1,2^. For fission to occur, dynamin-related protein 1 (Drp1) is recruited to the outer mitochondrial membrane for the constriction and separation of the organelle into two^3^. Fusion and fission are strategies for quality control, allowing for the distribution and turnover of mitochondrial components through the network, and facilitate cellular adaptation to different energetic demands^1,4^. Therefore, changes in mitochondrial morphology and function can severely impact the overall ability of specialized cells to function properly^5–7^. For example, stem cells commonly have punctate mitochondria with under-developed cristae, while highly specialized cells such as mature neurons and sperm have highly complex and elongated mitochondrial networks^8–10^.

**Figure 1:**
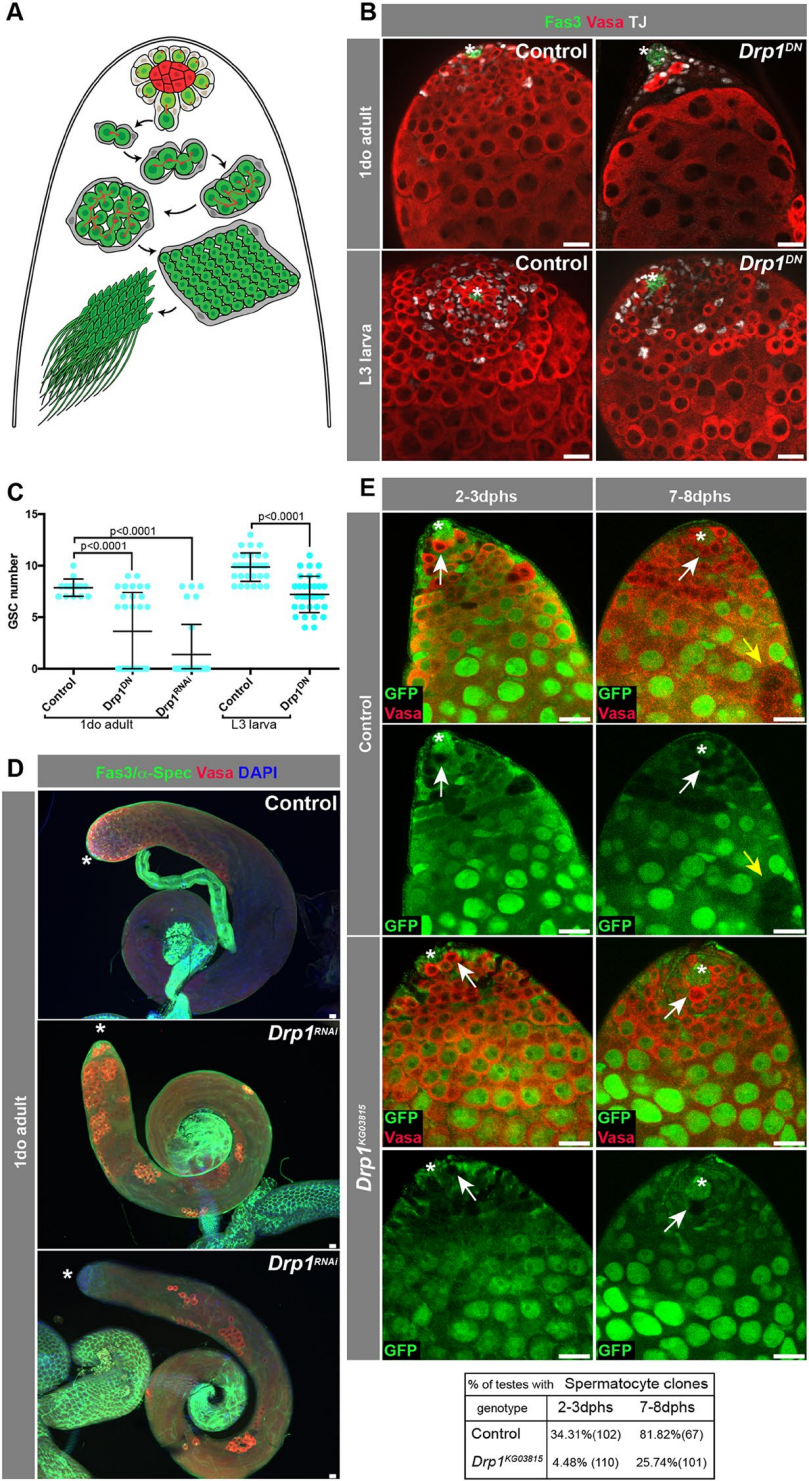
Drp1 is required for GSC maintenance in larval stages. (**A**) Schematic of the *Drosophila* testis. Hub cells (red) are surrounded by two stem cell populations: GSCs (in green) and CySCs (in gray). CySCs give rise to CCs that accompany the developing germline until spermiogenesis. GSCs divide to self-renew and give rise to a gonialblast, which undergoes four rounds of TA divisions prior to undergoing meiosis and terminal differentiation into sperm. (**B**) Representative immunofluorescence images of testes from ‘control’ (*nanosGAL4:VP16* > *w*^1118^) and ‘*Drp1*^*DN*^’ (*nanosGAL4:VP16* > *Drp1*^*DN*^) flies at 1 day old (do, post eclosion) or L3 larval stage. (**C**) Quantification of GSC number. Two-tailed t-test used. (**D**) Examples of testes from 1do animals. Note the range of distribution in the *nanosGAL4:VP16* > *Drp1*^*RNAi*^ animals (with and without GSCs). (**E**) Representative images of FRT-mediated clonal generation in control and *Drp1*^*KG03815*^ backgrounds. Clones are marked by the absence of GFP (see Methods). GSC clones are pointed by white arrows, while spermatocyte clones are pointed by yellow arrows. Quantification of clones at different time points (dphs, days post heat shock) displayed in adjacent table. GSC clones were quantified in^19^. In all images, asterisk (*) represents the hub; Scale bars, 20 μm. Individual images representative of >20 samples acquired from 3 biological replicates.

Spermatogenesis is the process by which male gametes are generated from a pool of undifferentiated stem/progenitor cells that undergo several rounds of transit-amplifying (TA) mitotic divisions, meiosis and terminal differentiation. The *Drosophila* testis is an excellent model for spermatogenesis studies due to the well conserved steps male germline stem cells (GSCs) undergo in order to become sperm^11–13^. In flies, a pool of about eight GSCs reside at the tip of the testis, surrounding a small group of cells, called hub cells, that secrete self-renewal factors necessary for stem cell maintenance^12^. GSCs divide to self-renew and give rise to a gonialblast (GB), which undergoes four rounds of TA divisions, prior to the expression of a set of spermatocyte-specific genes. At this stage, spermatocytes initiate a growth phase, followed by meiosis, to generate spermatids, and ultimately highly specialized sperm^12^. A population of somatic cells called cyst cells (CCs) develop in close association with the germ line. CCs are generated from a pool of somatic cyst stem cells (CySCs) that are present at the tip of the testis, adjacent to GSCs, and divide to maintain the CySC pool and generate a constant supply of cyst cells that ensure germ cell differentiation, similar to Sertoli cells in mammals^14^ (Fig. 1A).

Activation of epidermal growth factor receptor (EGFR) signaling in cyst cells is a critical mechanism that coordinates the maturation of soma and germ line^15–18^. Germ cells express and secrete the EGF-ligand Spitz, which binds to the EGFR and activates signaling in cyst cells. EGFR signaling promotes CC differentiation, which in turn governs, non-autonomously, synchronous spermatogonial TA divisions and spermatocyte differentiation^15–18^. The downregulation of EGFR signaling in cyst cells leads to the accumulation of germline cysts that divide asynchronously and fail to complete mitotic TA divisions^15–18^, while the hyperactivation of EGFR signaling results in bypassing mitotic TA divisions and premature transition to the spermatocyte stage^18^.

In a previous screen for factors that regulate mitochondrial dynamics and impact GSC maintenance in *Drosophila* testis, disruption of either mitochondrial fusion or fission resulted in a decrease in GSC number^19^. Mitochondrial fusion was shown to impact adult GSC maintenance through dysregulation of lipid metabolism in a cell-autonomous manner^19^. Similarly, disrupting fission throughout development by inhibiting Drp1 led to loss of GSCs in testes from 10 day-old (do) adult flies. However, in contrast, the generation of *Drp1*^−/−^ null GSCs in adults by FRT-mediated recombination indicated that *Drp1* is not absolutely required for GSC maintenance at this stage^19–22^. Furthermore, simultaneous disruption of fission and fusion did not rescue phenotypes resulting from disruption of fusion alone, suggesting that the loss of GSCs was not due exclusively to an imbalance in mitochondrial dynamics^19^. Therefore, we hypothesized that Drp1-mediated mitochondrial fission likely acts to maintain GSCs during development via a distinct mechanism. Here we show that fission of the mitochondrial network is required to suppress increases in reactive oxygen species (ROS) levels; elevation of ROS in germ cells leads to the activation of EGFR signaling in adjacent cyst cells, resulting in loss of GSCs and early spermatogonia due to premature differentiation.

## Results

### *Drp1* is required for the maintenance of spermatogonia in the larval testis

To further investigate a role for *Drp1* in the male germ line, a dominant-negative form of Drp1 (‘Drp1^DN^’) was expressed in GSCs and early spermatogonia using the *nanosGAL4:VP16* ‘driver’^23,24^, and testes were dissected from third instar (L3) larvae or 1-day old adults (Fig. 1B,C). In testes from 1-day old animals, the median number of GSCs was significantly lower than in control testes, with a number of samples completely lacking GSCs (Fig. 1C). Although complete loss of GSCs was not observed in larval testes, the number of GSCs was significantly decreased (Fig. 1B,C). A similar trend was obtained when *Drp1* was depleted in GSCs and early spermatogonia using a RNAi-mediated approach^25^ (Fig. 1C,D, note testes with complete absence of GSCs in D). As noted above, using the Flp/FRT site-directed recombination technology^20–22^, *Drp1*^−/−^ GSC progeny were generated from previously heterozygous cells (i.e., *Drp1*^−^*/Drp1*^+^, *tubulin-GFP*) and now harbor two copies of a *Drp1* null allele. These GSC ‘clones’ and their progeny are marked by the absence of GFP, which is present on the wild-type chromosome. These experiments showed that *Drp1* is not absolutely required autonomously for GSC maintenance in adults^19^, and that *Drp1*^−/−^ germ cells are capable of differentiation (Fig. 1E).

**Figure 2:**
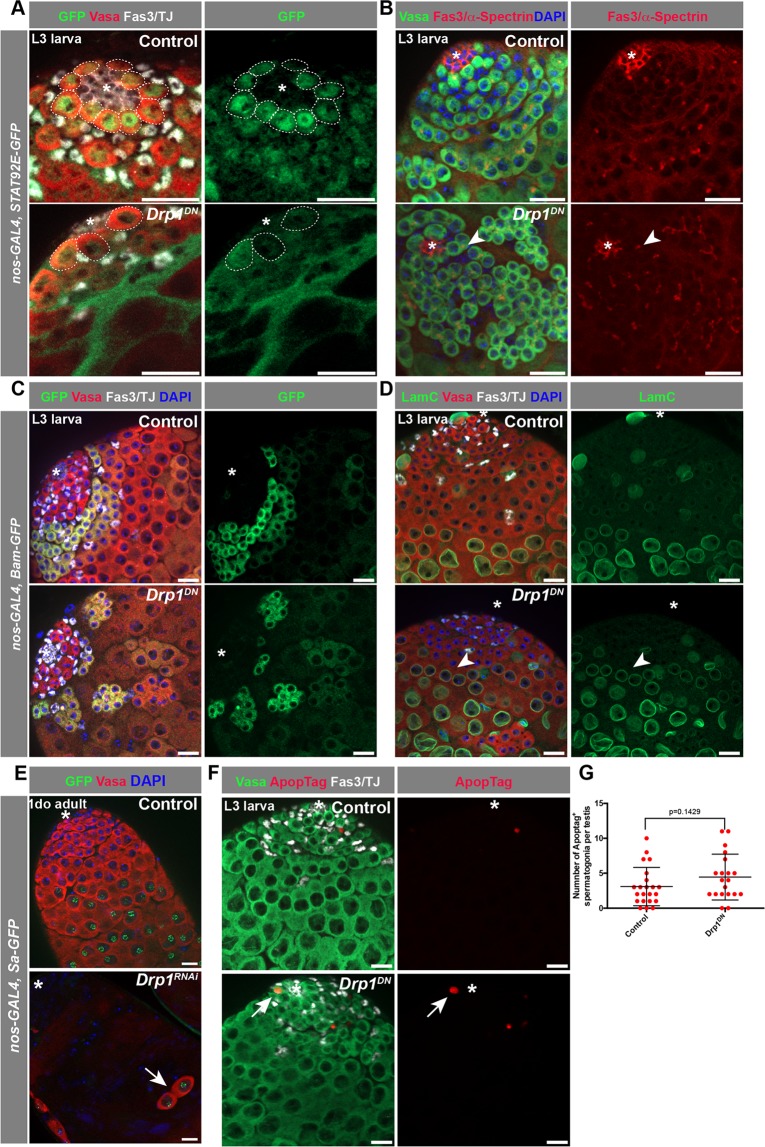
Disruption of Drp1 leads to spermatogonial loss. (**A**) Representative image of testes from L3 larvae expressing a *Stat92E:GFP* transgene. Dotted circles outline GSCs. (**B**) Examples of testes from control and *Drp1*^*DN*^ animals in which the progression of germline differentiation is observed with an antibody against the fusome marker α-Spectrin. Arrowhead notes barbell shaped fusome in a 2-cell cyst adjacent to the hub. (**C**) Images of L3 larval stage testes from animals carrying a *Bam:GFP* transgene, which marks (TA) spermatogonia. (**D**) Examples of testes from L3 larvae in which spermatocytes can be identified with an antibody against the lamin LamC. Note the high expression of LamC in spermatocytes (and in some somatic cells) in controls. Arrowhead points to germline cyst expressing high levels of LamC. (**E**) Representative images of testes from 1do adults expressing *Spermatocyte Arrest:GFP (Sa:GFP)*. Arrow points to 2-cell cyst positive for *Sa-GFP*. (**F**) Representative images of L3 larval testes stained for apoptotic cells with ApopTag. (**G**) Quantification of the number of ApopTag^+^ spermatogonia per testes. Two-tailed t-test used. In all images, asterisk (*) represents the hub; Scale bars, 20 μm. Individual images representative of >20 samples acquired from 3 biological replicates.

GSCs can be distinguished by their location adjacent to the hub, presence of a spherical spectrosome, an ER-like organelle, and accumulation of nuclear Signal Transducer and Activator of Transcription (STAT)^26,27^ (Fig. 2A). The progression of spermatogonial differentiation can be monitored by growth of the spectrosome into a branched fusome that connects the germ cells within the spermatogonial cyst. The fusome can easily be visualized via immunofluorescence microscopy using an antibody against the spectrosome/fusome marker α-spectrin^28^. When *Drp1* activity was blocked, germ cells in the GSC position displayed reduced levels of STAT accumulation in the nucleus (Fig. 2A) and ‘barbell’ shaped fusomes, rather than spherical spectrosomes (Fig. 2B). Early germline differentiation can also be determined by the expression and accumulation of Bag-of-marbles (Bam), a protein present in 4–16 cell spermatogonial cysts, which is required for cessation of TA divisions^29,30^. When *Drp1* activity was disrupted, fewer Bam^+^ spermatogonial cysts were observed and the normal spatio-temporal organization of differentiating germ cells was disrupted (Fig. 2C).

Early spermatocyte cysts can be identified by high expression of the nuclear lamin protein LamC^31^. Consistent with loss of early germ cells induced by disruption of *Drp1* function, in testes in which Drp1 activity was inhibited, later stage germline (i.e., spermatocyte) cysts with high levels of LamC were observed closer to the apical tip, when compared to testes from control animals (Fig. 2D**)**. Similarly, in testes from 1do adults, spermatogonial cysts containing 2–8 cells expressed the spermatocyte-specific marker *Spermatocyte arrest:GFP* (Sa:GFP) upon inhibition of *Drp1* activity^32^ (Fig. 2E), suggesting that these germline cysts are likely differentiating prematurely prior to completing TA divisions. These data are consistent with the observation that FRT-mediated *Drp1*^−/−^ null GSCs can give rise to spermatocytes (Fig. 1E). Importantly, no significant increase in apoptosis was observed in early spermatogonia when *Drp1*^*DN*^ was expressed (Fig. 2F–G). Together, these data suggest that loss of GSCs and early spermatogonia due to inhibition of *Drp1* activity is due, at least in part, to premature differentiation.

### Loss of Drp1-mediated mitochondrial fission results in increased ROS

**Figure 3:**
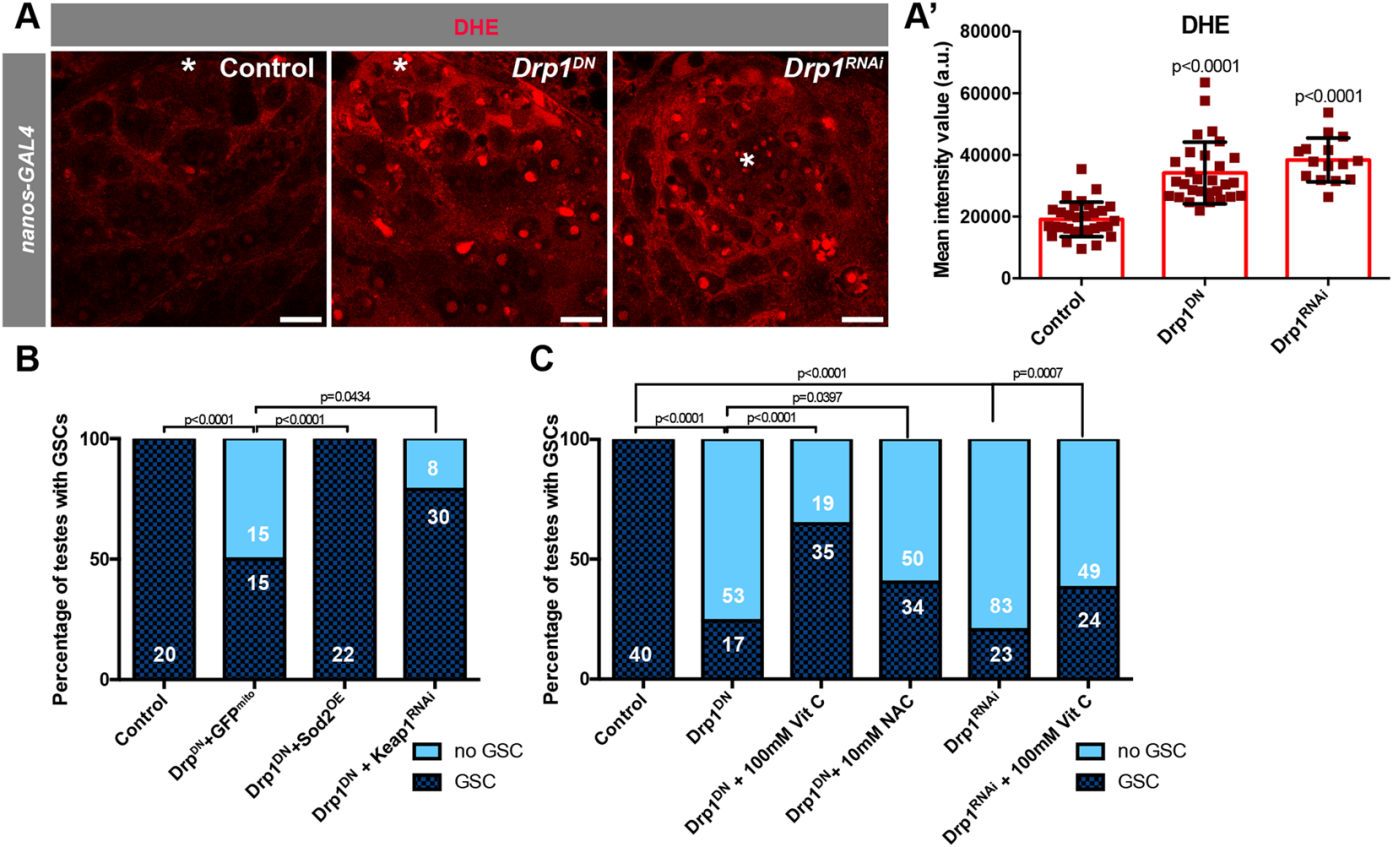
Disruption of mitochondrial fission results in high ROS levels. (**A)** Examples of L3-stage larval testes stained with the ROS-detecting dye DHE. (**A’**) Quantification of the intensity mean of the DHE signal in GSCs (see Methods). Two-tailed t-test used. (**B**,**C**) Representation of the percentage of testes with at least one GSC present at the niche versus no GSCs present in 1do animals (see Fig. 1D for examples). Number of testes analyzed in each category displayed on the graph in white. Note that GAL4-UAS levels were controlled for in C by the addition of a *UAS-GFP*^*mito*^ to *UAS-Drp1*^*DN*^. In C, *Drp1*^*DN*^ and *Drp1*^*RNAi*^ were the only UAS-based transgenes present. Two-sided fisher’s exact used. In all images, asterisk (*) represents the hub; Scale bars, 20 μm. Individual images representative of >20 samples acquired from 3 biological replicates.

To begin to elucidate the mechanism by which loss of Drp1 results in loss of early spermatogonia, mitochondrial morphology and activity were assessed. To validate that blocking Drp1 activity results in an overly fused mitochondrial network, a transgene harboring a mitochondrially-targeted GFP (GFP^mit^°) was expressed in GSCs and spermatogonia with the *nanosGAL4:VP16* ‘driver’. Though mitochondria in control early germ cells are predominantly small and punctate, highly fused mitochondria were observed in spermatogonia expressing *Drp1*^*DN*^ or *Drp1*^*RNAi*^ (Fig. S1A–C). Interestingly, with the co-expression of *GFP*^*mit*^*°*, a greater percentage of testes were obtained with spermatogonia remaining. The addition of another UAS-based transgene would decrease the amount of *Drp1*^*DN*^ or *Drp1*^*RNAi*^ produced, suggesting that Drp1 activity levels are indeed important for early germ cell maintenance. Fused mitochondrial networks are often correlated with organelles with considerable electron transport chain (ETC) activity, high oxidative phosphorylation capacity, and consequently, increased generation of reactive oxygen species (ROS)^1,2^. In order to assess the levels of cellular ROS, larval testes were stained with the dye dihydroethidium (DHE), which revealed that Drp1 inhibition caused a significant increase in ROS levels at the apical tip of the testis (Fig. 3A-A’).

In order to determine whether the increase in ROS contributes to GSC loss caused by the depletion of Drp1, genetic and pharmacological manipulations to dampen ROS levels were performed, while also depleting or blocking Drp1 function. Co-expression of the mitochondrial ROS scavenger superoxidase dismutase (*Sod2*) together with expression of *Drp1*^*DN*^ in GSCs and early spermatogonia suppressed the loss of GSCs due to the block in Drp1 activity (Fig. 3B). A similar trend was observed when an RNAi construct targeting *Keap1*, which prevents the activation of the anti-oxidant response mediated by Cnc/NRF2, was co-expressed with *Drp1*^*DN*^ (Fig. 3B). Additionally, treatment with either antioxidants Vitamin C (VitC) or N-acetylcysteine (NAC) partially suppressed GSC loss caused by the block in Drp1-mediated mitochondrial fission^33^ (Fig. 3C). Vitamin C also suppressed the loss of STAT accumulation in the nuclei of Drp1-inhibited cells in the GSC position (n = 69/84 STAT^+^ GSCs in *Drp1*^*DN*^ vs n = 69/70 STAT^+^ GSCs in *Drp1*^*DN*^ + VitC, p < 0.0001; n = 10 testes for each condition). Together, these data suggest that disruption in mitochondrial fission results in the increase in ROS production that is responsible for GSC loss.

### ROS-activated EGFR signaling in cyst cells contributes to spermatogonial loss

**Figure 4:**
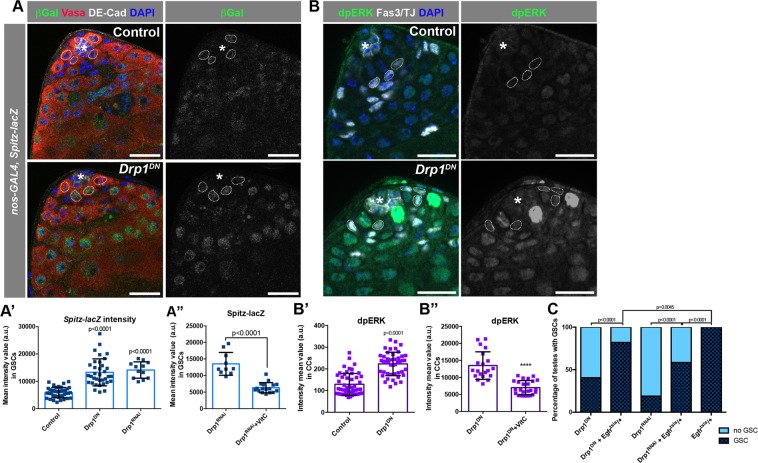
EGFR signaling is activated in cyst cells upon block in germline mitochondrial fission. (**A**) Representative images of testes from animals carrying a *Spitz-lacZ* transgene. Dotted circles indicate GSC nuclei, where measurements were taken. (**A’**) Quantification of the intensity mean of β-galactosidase expression in GSCs (see Methods). Two-tailed t-test used. (**A”**) Quantification of the intensity mean of β-galactosidase expression in GSCs from *nanosGAL4:VP16* > *Drp1*^*RNAi*^ flies raised either in regular diet or supplemented with Vitamin C. Two-tailed t-test used. (**B**) Representative images of testis tips stained with an antibody against diphospho-ERK (dpERK), an established readout for the EGFR pathway. Dotted circles indicate early CC nuclei, where measurements were taken. (**B’-B”**) Quantification of the intensity mean of dpERK stains in cyst cell nuclei (see Methods). Two-tailed t-test used. (**C**) Representation of the percentage of testes with at least one GSC present at the niche versus no GSCs present in 1do animals. Number of testes analyzed in each category displayed on the graph in white. Two-sided fisher’s exact used. For A and B, >20 samples acquired from 3 biological replicates. Asterisk (*) represents the hub; scale bar, 20 μm.

Recently, it was demonstrated that elevated ROS in germ cells can contribute to the activation of the EGFR signaling pathway in somatic cyst cells^33^. Upon an increase in ROS levels, the EGF-ligand *Spitz* was upregulated in germ cells, resulting in higher EGFR activation in the adjacent soma and, consequently, loss of GSCs and early spermatogonia due to premature differentiation^33^. To investigate whether EGFR signaling is increased when Drp1 activity is blocked in early germ cells, flies harboring a *Spitz-lacZ* transgene were used to investigate the levels of ligand expression^33^. When Drp1 function was inhibited, beta-galactosidase levels increased significantly in germ cells (Fig. 4A-A’). Consistent with the contribution of ROS to this phenotype, the increase in beta-galactosidase signal derived from *Spitz-*lacZ upon *Drp1* depletion was suppressed by treatment with Vitamin C (Fig. 4A”). Di-phosphorylation of ERK (dpERK) in somatic cyst cells can be used as a read-out of EGFR signaling and measured to assess levels of EGFR activation^18,34,35^. Consistent with an increase in the *Spitz* reporter, *Drp1* inhibition in spermatogonia also led to increased levels of dpERK in cyst cell nuclei (Fig. 4B-B’). The increase in dpERK was also partially suppressed by treatment with the antioxidant Vitamin C (Fig. 4B”), similar to the decrease in *Spitz-lacZ* expression, indicating that ROS plays an important role in the increase in EGFR signaling upon disruption of *Drp1* function. To test whether the increase in EGFR signaling contributes to the loss of GSCs caused by the block in Drp1 activity, a loss-of-function allele of *Egfr* (*Egfr*^*tsla*^) was used to decrease signaling concomitant with Drp1 inhibition in germ cells^36^. Under these conditions, reduction of EGFR significantly decreased the percentage of testes with no GSCs caused by Drp1 inhibition (Fig. 4C).

Although activation of Jun N-terminal kinase (JNK) signaling, another pathway known to be activated by ROS^37^, is also observed when Drp1 activity is blocked (Fig. S2A), decreasing JNK activity by removing one functional copy of the JNK homolog *basket* (*bsk*) did not rescue the loss of GSCs caused by the block of Drp1 activity^38,39^ (Fig. S2B). Altogether, these results suggest that disruption of mitochondrial fission results in an elevation of ROS in the testis, which in turn contributes to the activation of EGFR through upregulation of the ligand Spitz. The ectopic activation of EGFR signaling in cyst cells ultimately contributes to the loss of GSCs and early spermatogonia due to differentiation.

## Discussion

The data presented here highlight the role of mitochondrial fission in influencing spermatogonial behavior. Upon the loss of mitochondrial fission, GSCs and early spermatogonia were lost (Figs. 1B–D, 2A–C) due, at least in part, to differentiation (Fig. 2B,D–E). Mitochondrial fission appears important for keeping ROS levels in check, as the inhibition of *Drp1* activity led to increased ROS production (Fig. 3A-A’). Both the activation of the antioxidant response (Fig. 3B) and the pharmacological dampening of ROS levels (Fig. 3C) prevented the loss of GSCs caused by the inhibition of mitochondrial fission in GSCs and early spermatogonia. Mechanistically, the increase in ROS levels led to an increase in EGF ligand expression in early germ cells and the non-autonomous activation of EGFR signaling in adjacent somatic cyst cells (Fig. 4A-B”), which contributed to the loss of GSCs caused by *Drp1* inhibition (Fig. 4C).

Although ROS derived from the germline has been shown to activate EGFR in cyst cells^33^, it is likely that EGFR activation is induced via multiple mechanisms and not simply through the upregulation of germline *Spitz*. Importantly, previous findings have shown that the overexpression of secreted Spitz is not sufficient to robustly activate EGFR^18^ and suggested that EGFR levels may also need to be increased. In addition, ROS has been shown to activate EGFR by triggering autophosphorylation^40^, and downstream components of the EGFR signaling pathway, such as the MAPK cascade, could also be activated by ROS to increase signaling in these cells^40^. Finally, changes to the nuclear lamina can also affect EGFR signaling and niche organization in the *Drosophila* testis^31^. Alternatively, given the fact that activation of EGFR in CySCs and early cyst cells has been shown to increase their competitiveness for the niche^41^, GSCs in which Drp1 function was inhibited could be less fit leading to cyst cells with elevated EGFR/MAPK signaling outcompeting the germ cells for niche occupancy. Therefore, disruption of Drp1-controlled mitochondrial fission could affect GSC and early spermatogonial maintenance via EGFR signaling in multiple ways.

Our lab has demonstrated that mitochondrial fusion is required to maintain mitochondrial function in GSCs and that depletion of either *dMfn* or *Opa1* caused the accumulation of intracellular lipids and the activation of the Target of Rapamycin (TOR) pathway, ultimately resulting in GSC loss^19^. Interestingly, the block in mitochondrial fusion did not contribute to any obvious increase in ROS levels^19^. Though both mitochondrial fusion and fission are required for the maintenance of GSCs, our data indicate that different cellular response mechanisms are activated downstream of disrupted fusion versus fission. This model is supported by the fact that the concomitant disruption of mitochondrial fusion and fission did not rescue the loss of GSCs caused by either disruption alone^19^, suggesting that it is not simply the balance between fused and punctate mitochondria that governs cell fate in spermatogonia.

In summary, our results identify another mechanism through which mitochondria can influence cell fate. Mitochondrially-derived ROS can influence early spermatogonia to differentiate through the activation of the EGFR pathway in their neighboring somatic cyst cells. As mammalian spermatogonial progenitor cells are also closely influenced by adjacent stromal Sertoli cells^42^, and EGFR has been shown to influence mammalian germ cell differentiation^43^, it will be interesting to determine whether a similar relationship between mitochondrial fission, ROS accumulation and EGFR activation is also observed in mammals.

## Methods

### Fly husbandry and stocks

Flies were raised at 25 °C in a humidity controlled, light-dark cycle incubator (Percival), on standard cornmeal-molasses agar medium unless otherwise noted. The strains used were obtained from the following sources: Bloomington *Drosophila* Stock Center - *Stat92E:GFP* (BL38670), *UAS-Drp1*^*RNAi*^ (BL67160), *UAS-Sod2*.*Z* (*UAS-Sod2*, derived from BL27645), *cn*^*1*^ *Egfr*^*tsla*^ *bw*^*1*^*/CyO* (‘*Egfr*^*tsla*^’, derived from BL6501), *bsk*^*1*^ *cn*^*1*^ *bw*^*1*^ *sp*^*1*^ (‘*bsk*^*1*^’, BL3088), *bsk*^*2*^ *cn*^*1*^ *bw*^*1*^ *sp*^*1*^ (‘*bsk*^*2*^’, BL5283), *spitz-lacZ* (BL10462). Vienna *Drosophila* RNAi Center -. Personal gifts - *UAS-Drp1*^*K38A*.*HA*^ (‘*UAS-Drp1*^*DN*^’) (J. Chung), ‘*y*,*w*,*hs-flp*^*122*^*; Ubi-GFP FRT40A’*, *‘FRT40A’* and ‘*Drp-1*^*KG03815*^ *FRT40A’* (K. Mitra), *MMP1-lacZ* (U. Banerjee), *nos-Gal4::VP16* (M. Van Doren), *bam-GFP* (D. McKearin), *UAS-Keap1*^*RNAi*^ (H. Jasper), *sa-GFP* (M. Fuller). Lines not described here can be found in Flybase.

### Tissue-specific genetic manipulation

Early germ cell-specific gene expression was accomplished using the bipartite GAL4/UAS system. Flies carrying the *nosGAL4:VP16* transgene (‘driver’ expressed in GSCs and early germ cells) were crossed to *UAS-GFP*^*mit*^*°* to make a stock, and females were crossed to males harboring UAS-based transgenes. Control flies were the progeny of outcrosses from the GAL4 driver line to *w*^1118^ flies. To trigger RNAi response, short hairpin constructs were used to express double-stranded RNA targeting sequences complementary to the manipulated gene. When determining the genetic interaction between two UAS-based constructs, numbers of copies of UAS were taken into consideration. For example, the genotype for ‘Drp1^DN^’ in Fig. S1B is *UAS-GFP*^*mit*^*°/*+*;UAS-Drp1*^*DN*^*/nanosGAL4:VP16*, while ‘Drp1^DN^ + GFP^mito^’ in Fig. 3B is ‘*UAS-GFP*^*mito*^*/UAS-GFP*^*mito*^*;UAS-Drp1*^*DN*^*/nanosGAL4:VP16*’). Reporter elements, such as *Spitz-lacZ*, were combined into a stock with the *nosGAL4:VP16* ‘driver’ in order to make crosses as described above.

### GSC quantification

Antibodies against Vasa (germ cells) and Fas3 (hub) were used to count GSCs in dissected testes of 1-day old males (as a ‘final’ readout of development) or L3-stage larvae. GSCs are counted as Vasa^+^ cells in physical contact with the hub (Fas3^+^).

### FRT-mediated clonal analysis

Clonal analysis was performed by crossing *hs-FLP*^*122*^*; ubi-GFP FRT40A/Cyo* females to *drp1*^*KG03815*^ *FRT40A/CyO* or *FRT40A/CyO* males, selecting for the respective genotypes *hs-FLP/Y; drp1*^*KG03815*^ *FRT40A/ubi-GFP FRT40A* (for *drp1*^*KG03815*^ clone generation) or *hs-FLP/Y; FRT40A/ubi-GFP FRT40A* (for control clone generation). Selected male adults were heat-pulsed at 37 °C for 2 h for 2 consecutive days and analyzed 2–3 or 7–8 days post heat shock (dphs). Clones were selected by the absence of GFP antibody signal. In addition, Fas3 and Vasa antibodies were used to visualize the hub and germ cells, respectively. Testes samples were counted when at least one germ cell clone was observed.

### Genetic rescue experiments

All genetic rescue experiments were performed at 25 °C by crossing *UAS-GFP*^*mito*^*; nos-Gal4:VP16* females to *UAS-X; UAS-Drp1*^*DN*^ males (where “*UAS-X*” refers to the other UAS-based transgene used in the experiment). In order to control for GAL4 levels, a second *UAS-GFP*^*mito*^ was used in combination with *UAS-Drp1*^*DN*^. The percentage of testes with at least 1 GSC at the niche at 1 day post-eclosion was determined.

### Immunofluorescence and microscopy

Immunofluorescence (IF) was performed on whole-mount testes dissected in PBS and fixed in 4% paraformaldehyde (PFA) for 30 min (with the exception of dpERK stains, see below). After fixation, samples were washed twice for 15 min with 0.3% Sodium Deoxycholate in 1X PBS 0.3% Triton X-100 (PBST), followed by a 10 min wash with 0.1% PBST. Samples were blocked in 3% BSA in 0.1% PBST for 30 min prior to incubation overnight at 4 °C with primary antibodies unless otherwise noted. Primary antibodies used: mouse anti-Fas3 (7G10)(1:20), mouse anti-αSpectrin (3A9) (1:20), mouse anti-LamC (LC28.26)(1:20), rat anti-DE-Cad (DCAD2) (1:20) and mouse anti-βGal (40-1a)(1:20) were obtained from the Developmental Studies Hybridoma Bank; guinea pig anti-traffic jam (TJ) (1:3000, a gift from D. Godt), rabbit anti-Vasa (1:100, Santa Cruz); chicken anti-GFP (1:1000, Aves), and rabbit anti-phospho-p44/42 MAPK (1:100, Cell Signaling). Samples were incubated with secondary antibodies (1:500, Invitrogen) for 2 h at room temperature. Tissues were mounted in Vectashield with DAPI (Vector Laboratories).

All images were obtained using a Zeiss LSM 780 Laser Scanning confocal microscope or a Zeiss Observer.Z1 with Apotome 2 using the ZEN Black v2.0 software. Image processing, area measurement and pixel quantification were executed with ImageJ 1.50i (Wayne Rasband, National Institute of Health, http://imagej.nih.gov/ij).

### dpErk staining protocol and quantification

Fly testes were dissected into Schneider’s media supplemented with phosphatase inhibitor cocktail 2 (1:100, Sigma, cat#P5726). Dissected testes were then fixed for 30 min in 4% paraformaldehyde supplemented with phosphatase inhibitor, washed 4x in testis buffer (10 mM Tris-HCl pH6.8, 180 mM KCl) supplemented with phosphatase inhibitor, 0.2% BSA and 0.3% Triton TX100, prior to incubation in 1^o^ antibody overnight supplemented with phosphatase inhibitor. All samples were scanned under the same conditions (i.e., laser power, mounting slide, etc), and circular areas of 5334.834 nm diameter were used to acquire the fluorescence intensity of the dpERK signal in the somatic tissue surrounding early spermatogonia in the testis tips of the different genotypes analyzed.

### *Spitz-lacZ* quantification

All samples were scanned under the same conditions (i.e., laser power, mounting slide, etc), and circular areas of 5334.834 nm diameter were used to acquire the fluorescence intensity of the β-galactosidase signal in the nuclei of GSCs (with *nanos-GAL4:VP16*).

### DHE stains and quantification

For dihydroethidium (DHE) (Molecular Probes) stains, testes were dissected in Schneider’s medium and incubated with 30 μM of the dye for 5 min at room temperature prior to immediate mounting in Schneider’s medium and imaging. All samples were scanned under the same conditions (i.e., laser power, mounting slide, etc), and circular areas of 5334.834 nm diameter were used to acquire the fluorescence intensity of DHE signal in the nuclei of GSCs or the first onset of *bam-GAL4:VP16* expressing cells, accordingly (as determined by the expression of GFP^mito^).

### TUNEL assay

TUNEL assay was performed using ApopTag® Red *In Situ* Apoptosis Detection Kit (Millipore Sigma). Testes were dissected in 1x PBS and fixed in 2% PFA for 30 minutes. Samples were washed twice for 10 minutes each in 0.3% Sodium Deoxycholate 0.3% PBS-T and were then incubated in Equilibrium Buffer for 10 minutes. Samples were incubated at 37 °C for 1 hour in Reaction Buffer with Tdt enzyme in a 7:3 (buffer:enzyme) ratio. Samples were incubated for 10 minutes in Stop/Wash Solution and then washed in 0.1% PBS-T for 10 minutes. Samples were then transferred to diluted anti-DIG Rhodamine in Blocking Solution at a 47:53 ratio for 30 minutes, after which they were washed in 0.1% PBS-T twice for 10 minutes each and blocked in 3% BSA in 0.1% PBS-T. The aforementioned protocol for immunofluorescence was then resumed.

### Statistics

Quantitative experiments were evaluated for statistical significance using the software Graphpad Prism v6.0, after verifying the normality of values and equivalence of variances. For stem cell counts, fluorescence intensity mean values, tunnel assays, TA length estimation and fertility tests, means with standard deviations are displayed, and the statistical differences between mutant samples and controls were addressed using a Student’s two-tailed t-test. For the frequency of testes with GSCs, results were translated into individual contingency tables for each condition, where each row defines a genetic background (for example *Drp1*^*RNAi*^ vs control), each column defines an outcome (with or without GSCs) and each value is an exact count. Statistical significance was assayed using a two-sided Fisher’s exact test. In all cases, the specific statistical test used, along with numbers and significance, can be found in figure legends and/or figure panels.

## Supplementary information

Supplementary Figures

## Data Availability

The authors declare that the main data supporting the findings of this current study are available within the paper. All other data supporting the findings of this study are available from the corresponding author on reasonable request.
