# Supplementary material for: Mitochondrial fission regulates germ cell differentiation by suppressing ROS-mediated activation of Epidermal Growth Factor Signaling in the Drosophila larval testis
Source: Sci Rep. 2019 Dec 23;9:19695. doi: 10.1038/s41598-019-55728-0 (PMC6927965; doi:10.1038/s41598-019-55728-0)
Supplement: Supplementary file 1 — Supplementary Figures [file 41598_2019_55728_MOESM1_ESM.pdf]

Mitochondrial fission regulates germ cell differentiation by suppressing ROS-mediated activation of Epidermal Growth Factor Signaling in the *Drosophila* larval testis

Rafael Sênos Demarco[1] & D. Leanne Jones[1,2,3]

[1]Department of Molecular, Cell and Developmental Biology, [2]Molecular Biology Institute, [3]Eli and Edythe Broad Center of Regenerative Medicine and Stem Cell Research, University of California, Los Angeles, Los Angeles, CA 90095, USA

Supplemental Figure Legends

Figure S1. Depletion or inhibition of Drp1 results in hyperfused mitochondria

A-C. Representative images (>20 samples acquired from 3 biological replicates) of testes from 1do animals expressing GFP^{mito} in GSCs and early spermatogonia under the driver *nanosGAL4:VP16*. Mitochondrial fission was inhibited by expression of *$Drp1^{DN}$* (B) or *$Drp1^{RNAi}$* (C). Full genotypes: (A) *w^{1118}/Y; UAS-GFP^{mito}/+; nosGAL4:VP16/*+ (B) *w^{1118}/Y; UAS-GFP^{mito}/+; nosGAL4:VP16/UAS-$Drp1^{DN}$* (C) *w^{1118}/Y; UAS-GFP^{mito}/+; nosGAL4:VP16/UAS-$Drp1^{RNAi}$.* Asterisk (*) represents the hub; scale bar, 20μm.

Figure S2. JNK activation did not contribute to the GSC loss caused by inhibition of Drp1.

A. Images of testes from animals harboring the *MMP1-lacZ* transgene, a readout for the activation of the JNK pathway. Note the somatic activation of MMP1 in *nanosGAL4:VP16>$Drp1^{DN}$* animals. **B.** Representation of the percentage of testes with at least one GSC present at the niche/no GSC present in 1do animals. Number of testes in analyzed each category displayed on the graph in white. Two-sided fisher's exact used. In B, asterisk (*) represents the hub; scale bar, 20μm. Individual images representative of >20 samples acquired from 3 biological replicates.

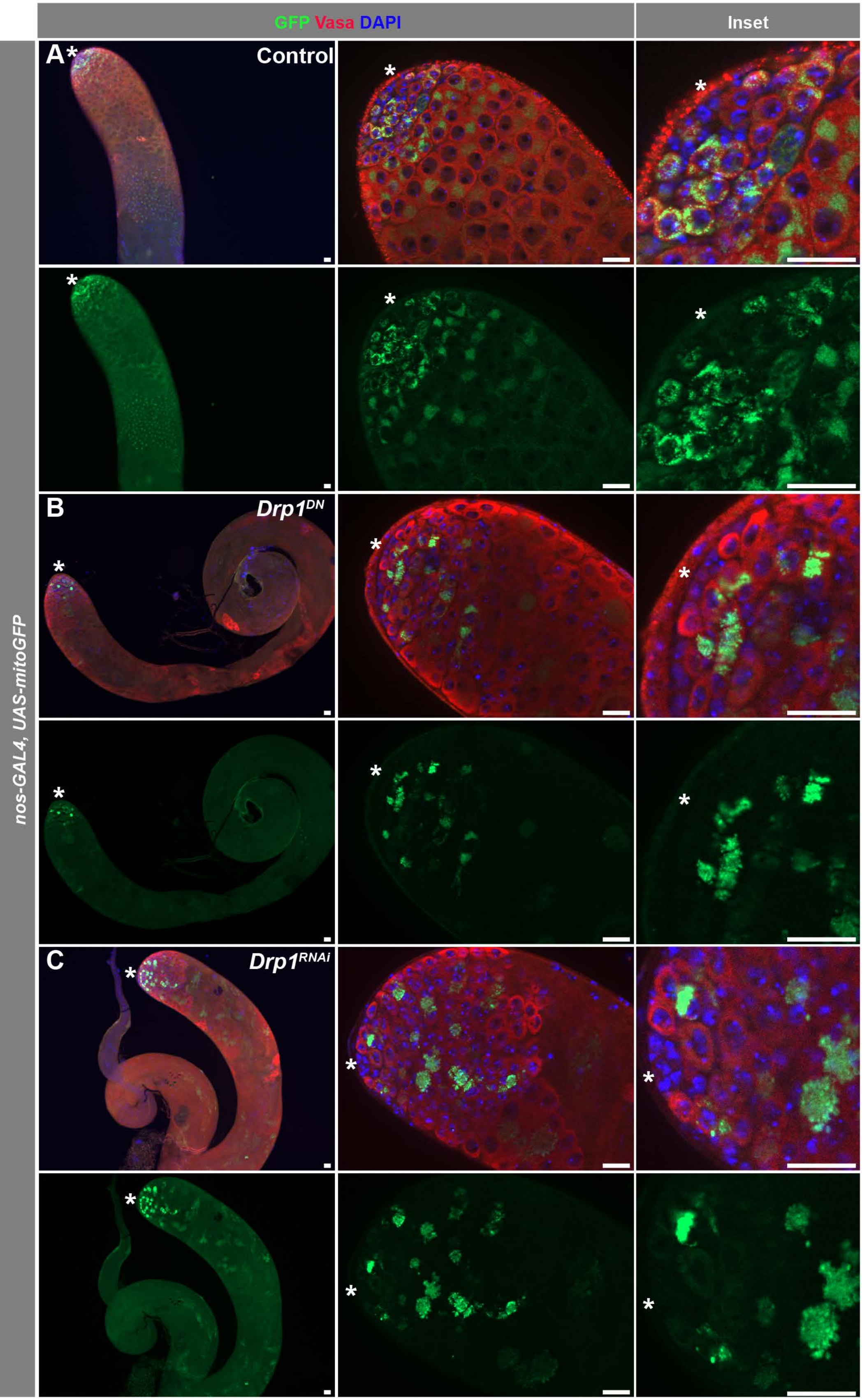

GFP Vasa DAPI
Inset
A
Control
B
Drp1^DN
C
Drp1^RNAi
nos-GAL4, UAS-mitoGFP

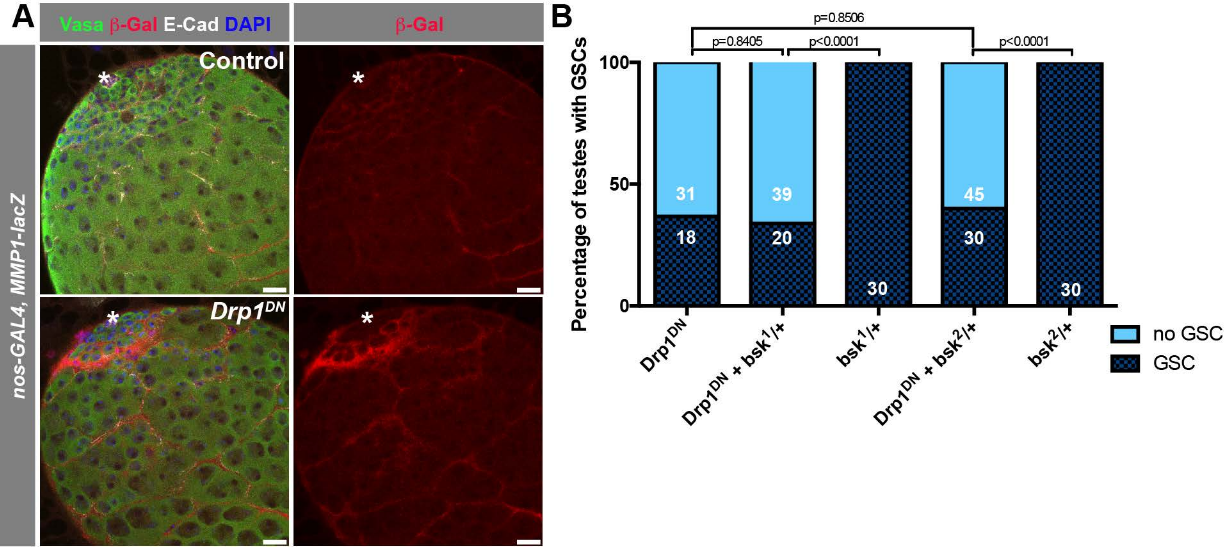

A
Vasa β-Gal E-Cad DAPI
β-Gal
nos-GAL4, MMP1-lacZ
Control
Drp1DN
B
p=0.8506
p=0.8405
p<0.0001
p<0.0001
Percentage of testes with GSCs
100
50
0
31
18
39
20
30
45
30
30
Drp1DN
Drp1DN + bsk1/+
bsk1/+
Drp1DN + bsk2/+
bsk2/+
no GSC
GSC
